# Indoleamines Impart Abiotic Stress Tolerance and Improve Reproductive Traits in Hazelnuts

**DOI:** 10.3390/plants12061233

**Published:** 2023-03-08

**Authors:** Murali-Mohan Ayyanath, Mukund R. Shukla, Praveen K. Saxena

**Affiliations:** Department of Plant Agriculture, Gosling Research Institute for Plant Preservation, University of Guelph, Guelph, ON N1G 2W1, Canada

**Keywords:** *Corylus* sp., flowering, tryptophan, serotonin, adaptation, biochemical marker

## Abstract

Hazelnuts have recently gathered tremendous attention due to the expansion of the confectionary industry. However, the sourced cultivars fail to perform in initial phase of cultivation as they enter bare survival mode due to changes in climatic zones, for example, Southern Ontario, where the climate is continental, as opposed to the milder climate in Europe and Turkey. Indoleamines have been shown to counter abiotic stress and modulate vegetative and reproductive development of plants. Here, we examined the effect of indoleamines on the flowering response of the dormant stem cuttings of sourced hazelnut cultivars in controlled environment chambers. The stem cuttings were exposed to sudden summer-like conditions (abiotic stress) and the female flower development was assessed in relation to endogenous indoleamine titers. The sourced cultivars responded well to serotonin treatment by producing more flowers compared to the controls or other treatments. The probability of buds resulting in female flowers was highest in the middle region of the stem cuttings. It is interesting to note that the tryptamine titers of the locally adapted, and *N*-acetyl serotonin titers of native hazelnut cultivars, provided the best explanation for adaptation to the stress environment. Titers of both compounds were compromised in the sourced cultivars which resorted mostly to serotonin concentrations to counter the stress. The indoleamines tool kit identified in this study could be deployed in assessing cultivars for stress adaptation attributes.

## 1. Introduction

Hazelnut is a monoecious species with female and male reproductive structures on the same branch that do not mature synchronously [1]. Reproductive organs such as catkins are short-lived and sensitive to frost and fluctuations between warm and cold weather, which are quite common during early spring in Ontario [1]. Such climate is exceptionally damaging for commonly cultivated European cultivars of hazelnut (*Corylus avellana* L.) and may lead to rapid pollen dehiscence [2,3] besides a decline in female flower ratio in the sourced cultivars that are horticulturally important for the growers.

Indoleamine compounds, such as melatonin (MEL) and serotonin (SER), are known to play a vital role in alleviating abiotic stress, while improving growth parameters [4]. The indoleamines have recently emerged as highly efficient stress-mitigating compounds in plants. The role of melatonin in the regulation of plant morphogenesis, reproduction, and stress survival is now well documented. Both MEL and SER can function as antioxidants and plant growth regulators in improving plant survival and growth under stress environments, as shown earlier with real-time tracking of Quantum Dot nanoparticles of MEL and SER in the living tissues [5]. MEL mediated improvements in stress survival have additionally been documented in a diversity of species, where MEL functions to regulate diverse PGR biosynthetic pathways and signaling and defense networks, to mitigate the effects of cold stress [6]. Exposure to MEL was shown to up-regulate the expression of several cold-responsive and cold stress-related transcription factors (for example, CBF/DREBs, COR15a, CAMTA1 and ZAT10/12) in *Arabidopsis thaliana* in response to cold stress [6].

Tryptophan (TRP) is an essential aromatic amino acid that is one of the building blocks for protein synthesis and the source of many important compounds, particularly MEL and nicotinamide adenine dinucleotide in animals [7], and IAA and phytoalexins in plants [8]. In plants, TRP has been shown to affect various long-term physiological processes such as organ growth [9], responses to stress and pathogen elicitors [8], and tolerance of heavy metals [10]. Treatments with TRP as a foliar spray, seed priming, and soil application [11] have been found to increase growth, yield, physiological attributes, and nutrient content of produce in different crops. TRP may serve as a point of cross-talk among the MEL, auxin, and kynurenine pathways leading to a complex symphonic fine-tuning of plant development and adaptations. It has been noted that SER and Kynurenine compounds are abundant in plants, although in minute quantities compared to TRP. In *Catharanthus roseus*, the application of TRP improved growth and photosynthetic capacity, possibly due to the modulation of cytokinin levels in a dose-dependent manner, as well as increasing auxin (IAA), gibberellic acid, and abscisic acid levels [12]. However, soil-applied TRP is reported to be metabolized into many products including SER and auxin by the microbial population in the rhizosphere [13]. Phytohormones present in the soil affect plant growth and development and the phenotypic character of the soil microbiota has more of an influence on phytohormone production than the physicochemical properties of the soil [14], which would improve organoleptic traits. Interestingly, the reduction of stress-induced oxidative damage by MEL supplementation by the endophytic microbes was associated with the downregulation of the MEL biosynthetic pathway in grapevine plantlets [15,16], confirming the role of MEL supplementation in alleviating abiotic stress in plants. This sort of resource allocation could be used towards an improved nut set with added benefits in the form of improved organoleptic traits. Previous reports have found a variation in the phytohormones profile in reproductive structures of the plants under abiotic [17] and biotic [18] stresses. Biotic stress can trigger an imbalance of phytohormones, with as much as 140-fold IAA accumulation in infected flowers [18].

Stem cuttings of various hazelnut cultivars were assessed for cold tolerance and associated modulation of the physiology [3] of vegetative and reproductive parts. Lately, we noted that water percolation [19] during late winter was the key to triggering phenological and vital reproductive events that remained inactive during peak winter [20]. However, reproductive physiological events were seldom assessed by exposing dormant reproductive organs to constant sudden summer-like conditions, thus subjecting them to abiotic stress that could potentially be alleviated under the influence of indoleamines. Recent research has demonstrated that indoleamines may modulate plant responses via a symphony of actions of molecules in the entire indoleamine pathway rather than a specific molecule [5]. We hypothesized that abiotic stress tolerance in hazelnuts may be enhanced, through targeted application of the precursors and intermediate indoleamine compounds to enhance the pathway-based promotion of stress tolerance.

Indoleamines are commonly synthesized from TRP via its conversion to TRM (Tryptamine), followed by TRM to SER, NAS (*N*-acetyl serotonin), leading to MEL. The phytochemical mechanisms underlying megaspore and microspore development in hazelnuts, and their unique survival under fluctuating spring temperatures, are yet poorly understood. To test our hypothesis, exogenous application of TRP was conducted on the stem cuttings from locally bred and introduced/sourced cultivars where phenological events would be subjected to biochemical analyses to validate the proof-of-concept. Here, we predicted that biochemical characterization of the indoleamine pathway concerning growth and survival during fluctuating cold stress will lead to the development of a biochemical tool kit that might assist in evaluating desirable hazelnut genotypes suited to the continental climate of Ontario, Canada. In this study, we hypothesized that the stem cuttings of hazelnut cultivars would sustain the abiotic stress with improved female flower ratio when exposed to TRP or SER and the titers of TRP and metabolites would correspond to stress and flowering patterns. The findings from this research may facilitate the assessment of cultivars for stress tolerance and also improve stress mitigation strategies in crop production.

## 2. Results

Abiotic stress (thermal stress), experienced as poor performances in sourced cultivars of hazelnut, was studied by exposing stem cuttings from dormant trees to sudden summer-like conditions, with or without exogenous treatment of the ‘stress buster’ molecules, TRP and SER. The flowering response and associated titers of indoleamines were chosen as endpoints.

### 2.1. Preliminary Experiments to Determine Zeitgeber

**Figure 1:**
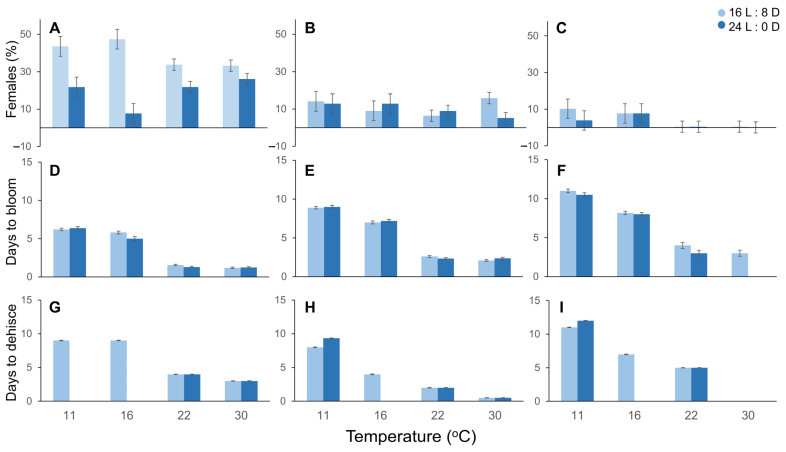
Effect of temperature and photoperiod on the percentage of female flowers, days to full bloom and days to pollen dehiscence in three hazelnut cultivars. ‘Slate’ (**A**,**D**,**G**) is known as a pollinizer and ‘Geneva’ (**B**,**E**,**H**) is a locally adapted cultivar. ‘Clark’ (**C**,**F**,**I**) has been sourced from Oregon State University, USA. Four temperatures (11, 16, 22 and 30 °C) and two photoperiod combinations (16 L (light blue bars) and 24 L (dark blue bars)) were tested. Bars represent means ± SEM.

In controlled condition experiments, the female flower ratio was affected by the interactions between selection, temperature, and photoperiod (*P* = 0.02) (Figure 1). Higher female ratios occurred when the stem cuttings were exposed to 16: 8 (L:D) (19%) rather than 24: 0 (11%). The duration needed to full bloom was affected by the interactions between temperature and photoperiod (*P* < 0.05). Buds that resulted in flowers reaching full bloom in 8.66 d, 6.86 d and 2.46 d, when exposed to 11, 16 and 22 °C respectively, but not at 30 °C, where a higher percentage of vegetative buds were observed. Pollen dehiscence was affected by the interactions between selection, temperature, and photoperiod (*P* < 0.05). Due to contrasting differences among the selections, the catkin study was not included in further experiments. For example, ‘Geneva’ dehisced pollen in 0.5 d when exposed to 30 °C, irrespective of the photoperiod, and ‘Clark’ dehisced pollen in 12 d when exposed to 11 °C and 24:0 h photoperiod conditions. Photoperiod (16:8 (L:D)) and temperature (22 °C) settings, derived from these preliminary experiments were used for investigating the effects of TRP and SER treatments.

### 2.2. Effect of TRP and SER Treatments on Hazelnut Bud Responses

**Figure 2:**
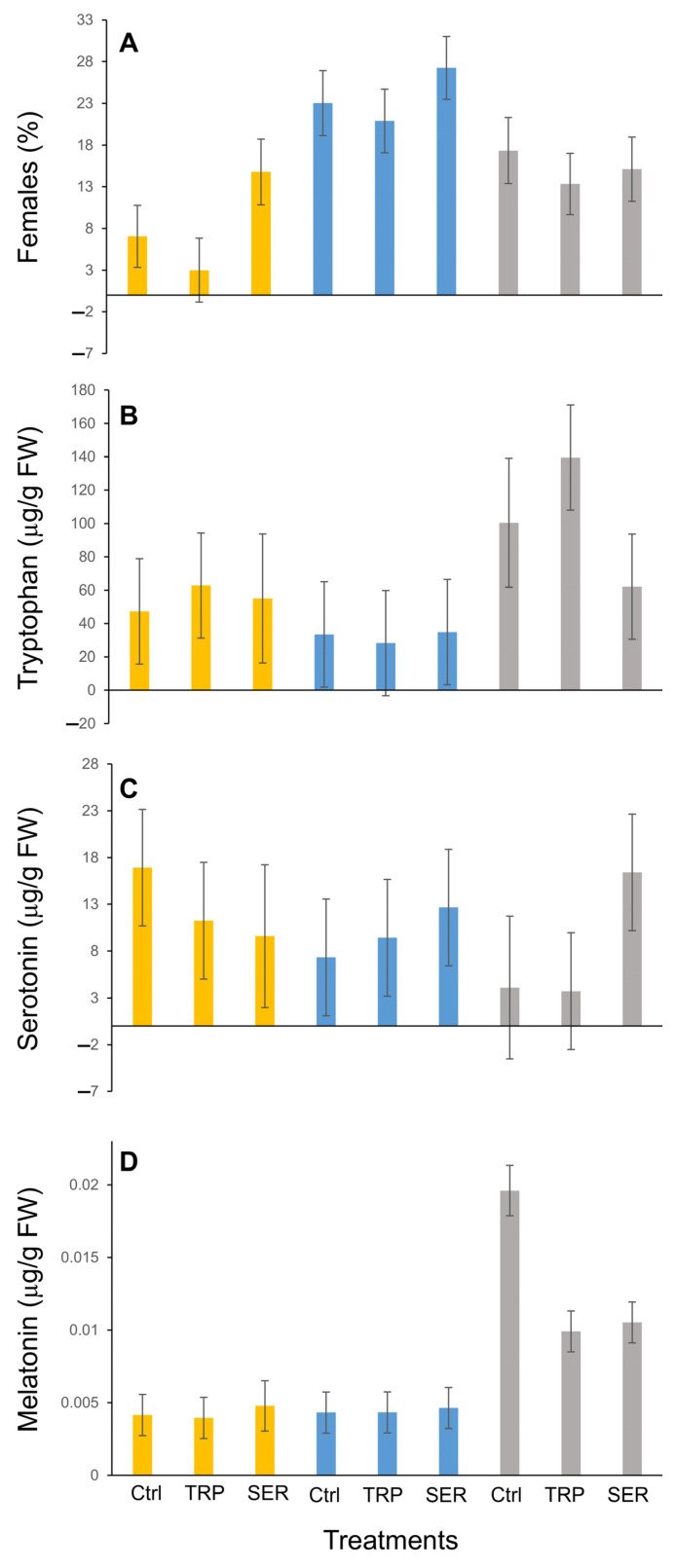
Effect of abiotic stress on flowering (**A**) and indoleamine metabolite concentrations *viz* tryptophan (**B**), serotonin (**C**), and melatonin (**D**) in dormant, vegetative and flower buds of stem cuttings from sourced (‘Delta’ yellow bars), locally adapted (‘Alex’ blue bars) and native hazelnuts (grey bars) when exposed to controls (water ‘Ctrl’), tryptophan (TRP, 100 μM) and serotonin (SER, 100 μM) treatments. Bars represent means ± SEM (µg/g FW) for that compound.

The interaction between selection (locally adapted ‘Alex’, sourced ‘Delta’ or natives) and the treatments (water, TRP or SER) was not statistically significant when vegetative and flower buds were combined to analyze the effects of TRP and SER concentrations using the UPLC-MS (*P*_(4,16)_ = 0.73 and 0.66, respectively) (Figure 2). However, when MEL concentrations were analyzed for similar parameters, a statistical significance was noted (*P*_(4,16)_ = 0.02). The native hazelnuts possessed the highest concentration of MEL compared to a locally adapted or sourced cultivar that contained about a fourth of the concentration. Among hazelnut cultivars, the treatments (TRP or SER) resulted in lowered MEL concentration by 33% compared to water (control). Due to varied responses in flowering patterns among the selections exposed to the treatments, the nature (kind) of the bud and assessment of the indoleamine pathway were deemed important for further analyses.

### 2.3. Effect of TRP and SER Treatments on Hazelnut Phenology

**Figure 3:**
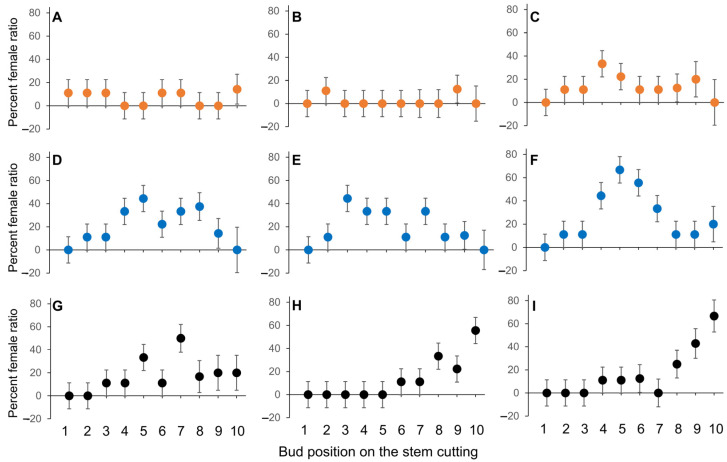
Bud position and probability of a female flower occurrence (female percentage) when the stem cuttings from sourced (‘Delta’ yellow dots), locally adapted (‘Alex’ blue dots) and native hazelnuts (black dots) were exposed to the control (water (**A**,**D**,**G**)), tryptophan (TRP 100 μM; (**B**,**E**,**H**)) and serotonin (SER 100 μM; (**C**,**F**,**I**)) treatments.

There were no statistical differences among the interactions of the position of the bud on the twig, the selection of hazelnut and the treatment (*P*_(36,661)_ = 0.52) (Figure 3). However, trends were noted among the interactions. All buds had either become a flower, that later turned into vegetative, or remained vegetative when these selections were exposed to sudden summer-like conditions (abiotic stress). ‘Alex’ possessed the highest number of flowers and ‘Delta’ the least. When these stem cuttings were exposed to water, buds positioned in the middle of the stem cuttings of ‘Alex’ and native hazelnuts mostly resulted in flowers, while buds of ‘Delta’ did not show clear trends. When stem cuttings were exposed to SER, a significant increase in the percentage of female flowers was noted in the buds positioned in the middle of the stem cuttings of ‘Alex’ and ‘Delta’ (Figure 3C,F,I). Interestingly, basal buds of native hazelnuts showed a steep increase in female percentage. Meagre pronounced trends were noted among the selections when the stem cuttings of ‘Alex’ and ‘Delta’ were exposed to TRP (Figure 3B,E,H). Native hazelnuts responded with a similar trend to SER treatment when exposed to TRP (Figure 3G–I).

### 2.4. Phytochemical Analyses by UPLC-MS

#### 2.4.1. Effect of TRP and SER Treatments on TRP

**Figure 4:**
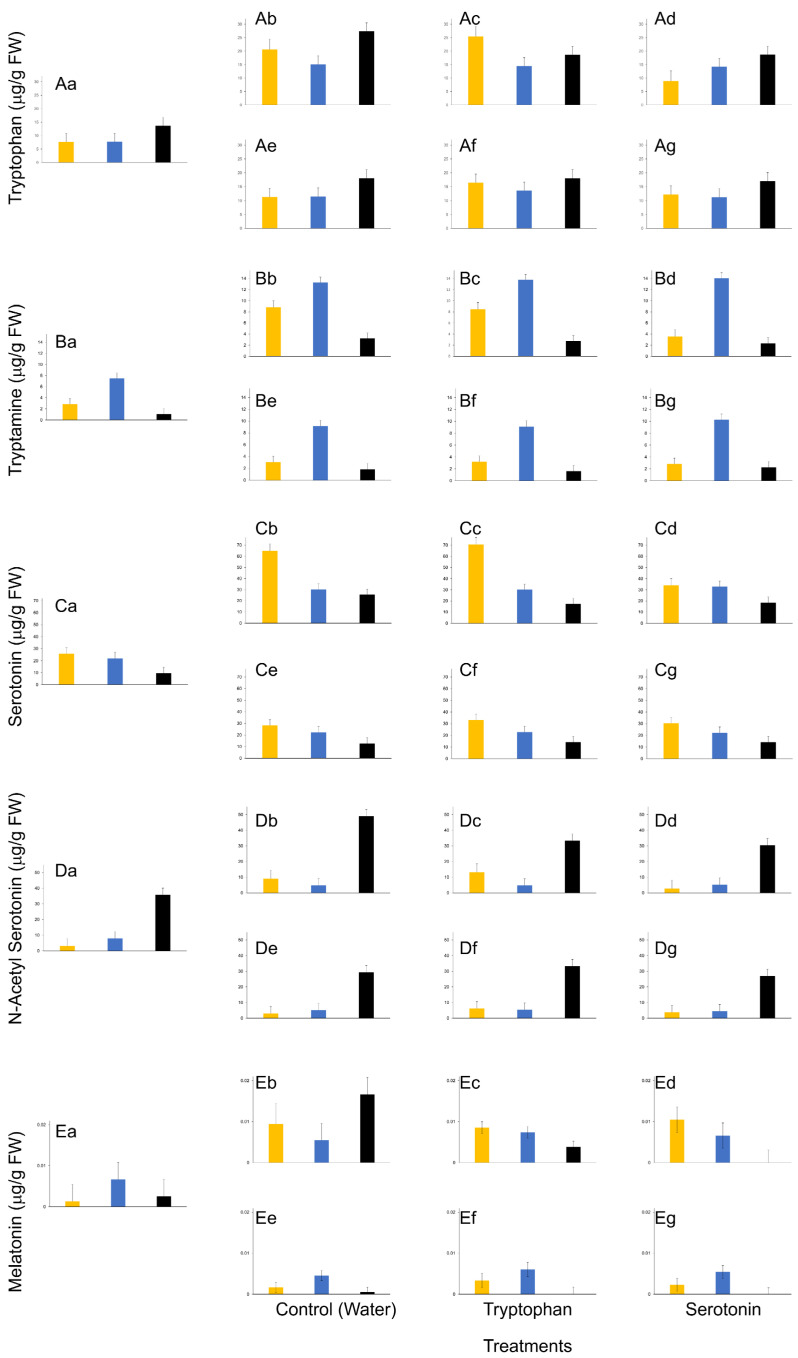
Effect of abiotic stress on indoleamine metabolite concentrations *viz* tryptophan (**A**), tryptamine (**B**), serotonin (**C**), *N*-acetyl serotonin (**D**), and melatonin (**E**) in dormant (**a**), flower (**b**–**d**) and vegetative (**e**–**g**) buds of stem cuttings from sourced (‘Delta’ yellow bars), locally adapted (‘Alex’ blue bars) and native hazelnuts (black bars) when exposed to the control (water), tryptophan and serotonin treatments. Bars represent means ± SEM (µg/g FW) for that compound.

Among the cultivars, when TRP concentrations were analyzed for the effects of treatments (SER or TRP), and kind of bud (flower or vegetative), no statistical significance was noted in the interactions (*P*_(4,39)_ = 0.4659) (Figure 4(Aa–Ag)). The effect of treatment on TRP concentration was near statistical significance (*P*_(2,39)_ = 0.0697), whereas the effects on the kind of bud and cultivar, were statistically significant (*P* < 0.05). In the dormant buds, TRP concentrations were noted to be similar in locally adapted and sourced cultivars but double in the natives. The trends remained similar if the resulting bud was vegetative in nature. However, TRP concentrations increased in the flowers of sourced cultivars when the dormant stem cuttings were exposed to water or TRP, but not SER. It is noteworthy that the concentrations of TRP in the flowers of the sourced cultivar, when exposed to SER and dormant buds, were similar. Also, it is interesting to note the unchanged concentrations of TRP among the flowers of locally adapted cultivars exposed to any of the treatments.

#### 2.4.2. Effect of TRP and SER Treatments on TRM

There were no statistically significant differences in the concentrations of TRM among the interactions of cultivars, treatment, and the kind of bud (*P*_(4,39)_ = 0.5257) (Figure 4(Ba–Bg)). The effects on the kind of bud and cultivar were statistically significant (*P* < 0.05), but not the treatment (*P* > 0.05). Dormant stem cuttings of the sourced cultivar, when exposed to SER, appeared to deter a change in TRM concentration in flowers. In all other cases, the TRM concentrations were not affected, irrespective of the cultivar, treatment, or kind of bud. It is interesting to note the changes in concentrations of TRM in buds of locally adapted cultivars, in comparison with TRP, where dormant buds possessed about half the concentration of TRM when compared to flowers.

#### 2.4.3. Effect of TRP and SER Treatments on SER

The interactions among the kind of bud, cultivar and treatment were nearly statistically significant when SER concentrations were analyzed (*P*_(4,39)_ = 0.0751) (Figure 4(Ca–Cg)). The concentrations of SER were affected by the kind of bud and cultivar (*P* < 0.05), but marginal statistical significance was noted in the effect of treatment (*P*_(2,39)_ = 0.1012). It is noteworthy that the concentrations of SER in the flowers of sourced cultivars when dormant stem cuttings were exposed to SER treatment in controlled conditions were unaltered. The concentration of SER increased in the flowers of locally adapted cultivars in comparison to their vegetative buds where the concentration of SER remained unaltered since dormancy. A similar trend was noted in the native hazelnuts, but the treatments (SER and TRP) suppressed the concentrations of SER in flower samples.

#### 2.4.4. Effect of TRP and SER Treatments on NAS

There were no statistically significant differences in the interactions among the kind of bud, cultivar, and treatment when NAS concentrations in the samples were analyzed (*P*_(4,39)_ = 0.3932) (Figure 4(Da–Dg)). There were statistically significant differences in the concentrations of NAS among the cultivars (*P*_(2,39)_ < 0.05). Trends remained similar to those observed for other metabolites in the flower samples of sourced cultivars, where SER treatment suppressed NAS concentrations. However, native hazelnuts seemed to have about a 3-fold higher concentration of NAS in dormant samples compared with other cultivars and this further increased (2-fold) in flower samples of stem cuttings that were exposed to control treatment. Also, an unaltered NAS titer among the samples of locally adapted cultivars was noteworthy.

#### 2.4.5. Effect of TRP and SER Treatments on MEL

There were no statistically significant interactions among the kind of bud, cultivar, and treatment when MEL concentrations from various bud samples were analyzed (*P*_(4,39)_ = 0.6004) (Figure 4(Ea–Eg)). However, the effect on MEL concentrations, due to the kind of bud, was statistically significant (*P* < 0.05). There was a trend where the dormant buds of locally adapted cultivars had higher titers of MEL compared to sourced and native hazelnuts. Furthermore, this trend continued into the vegetative bud stage but not into the flower stage. The higher titers of MEL in the locally adapted cultivars suggest that these plants may have a greater capacity for stress tolerance and adaptation compared to the sourced and native hazelnuts. In controls, the locally adapted cultivars possessed lower titers in comparison to the sourced or native hazelnuts. However, SER treatment decreased the titers in natives but was unaffected in locally adapted or sourced cultivars (Figure 4(Ee,Ef)). On the other hand, the TRP treatment resulted in similar trends as for the SER treatment, but the titers of MEL in the native hazelnut stem cuttings decreased nominally in comparison to controls.

## 3. Discussion

The responses of plants to fluctuating conditions are quite remarkable, especially in temperate trees where quiescence is often followed by flowering. Such trees quickly adapt to erratic temperature fluctuations by reallocating resources, for instance, choosing survival as opposed to the normal development. Indoleamines, melatonin (MEL) and serotonin (SER) have been found to counter stress by localizing and dispersing across cells that are exposed to disruptive thermal stresses [5]. This proof of concept, generated using the in vitro culture model (St. John’s wort) [5] was extrapolated into a laboratory setting before adapting it to field conditions [5]. Deploying a plethora of combinations, controlled conditioned experiments were conducted on hazelnut stem cuttings to understand the effects of indoleamines during thermal stress. The findings from this research provide novel insights into the mediation of abiotic stress by compounds of the indoleamine pathway, such as the broadly studied SER and MEL and mostly undetermined tryptamine (TRM) and NAS. These molecules could assist adaptation of young plants to new conditions besides alleviating environmental stresses in mature plantations via exogenous applications.

It is interesting to note that both photoperiod and temperature affected the female flower ratio among the cultivars. The treatments were effective in lowering the concentrations of MEL, compared to water, while improving flowering. For example, in datura, lower melatonin resulted in higher flowering [17].

Stem cuttings from dormant hazelnut cultivars, when exposed to sudden summer-like conditions (abiotic stress), had the least pronounced vegetative growth where no bud was adversely affected or died. Photoperiod affected the female flower ratio in controlled conditions. In nature, this ratio seems to be conditioned by the selection’s genetics and/or a combination of abiotic factors. Anecdotally, it is noted that every bud has the complete gear to become a female flower but only ca. 30% turn into female flowers. These female buds and the catkins (male) are differentiated during late spring (May–June) of the previous year. These events occur even before the nuts of the new season are observed on the branches. Unlike photoperiod, higher temperature appeared to have adversely affected the time to full bloom and pollen dehiscence in controlled conditions. Abiotic stress was countered by a decreased percentage of buds resulting in flowers before vegetative growth, especially in the sourced cultivar ‘Clark’. Stunted catkins, because of abiotic stress, suggest depletion of residual moisture in the catkin, when the stem cutting was exposed to extreme summer-like conditions, rendering male flowers sensitive to erratic springtime fluctuations. It was evident in ‘Clark’, a proven cultivar sourced from Pacific Northwest, in comparison to locally adapted ‘Geneva’, selected for nuts, and a pollinizer, ‘Slate’. Taken together, lower photoperiod and cooler temperatures appeared to be the appropriate zeitgebers for experiments with indoleamine treatments.

Plant stress amelioration by indoleamines has opened new avenues for improving the cultivation of horticulturally important trees such as apples, grapes, cherries, hazelnut etc. [21]. Climate crisis-borne erratic temperature fluctuations are particularly detrimental during catkin development and pollen dehiscence and often result in a decreased female flower ratio, especially in preferred cultivars sourced from a different climatic zone [1]. To improve the female flower percentage during abiotic stress (thermal stress—‘springtime cold fluctuations’ in field conditions and ‘sudden summer-like hot’ in controlled conditions), the exogenous applications of the indoleamines metabolite (SER) or their biosynthetic precursor TRP were administered. When dormant stem cutting of a sourced cultivar ‘Delta’ was exposed to SER, there was an increase in the female flower ratio compared to the control (water). However, the titers of TRP or indoleamine metabolites did not suggest any patterns or correlations with that of flowering. Natives responded well to the treatments via accumulation of TRP and SER titers when exposed to TRP and SER treatments, respectively, and a decrease in MEL titers compared to controls, suggested a negative feedback or regulatory effect of MEL on the indoleamine pathway [22]. However, flowering was unaffected due to treatments in the natives. Locally adapted ‘Alex’ phenotypically responded to SER treatment with marginally improved flowering, but TRP or metabolite titers mostly remained unaffected by the treatments. These findings may assist in designing experiments where exogenously applied TRP (foliar or drench) is used to prepare the sourced trees for the erratic fluctuations during early spring.

Simulating drenching treatment, when stem cuttings were exposed to either control (water) or treatments (TRP, SER), the fate of the top 10 apical buds, as hypothesized, had a higher female flower ratio irrespective of the cultivar chosen, but only in the SER treatment. Translocation of SER could have influenced the bud position from which a female flower resulted. The decreased female percentage corresponded with controls when stem cuttings were exposed to TRP. The molecule could have been deployed for stress protection as opposed to reproduction, or translocation and assimilation of TRP were slower than the phenological events. The earlier reasoning appears to agree with the titers of its metabolites, especially that of TRM. The titers of TRP did not differ significantly among the cultivars when dormant bud samples were assessed, except in natives where it was almost double, suggesting an adaptation strategy in these trees. Titers of TRP in root exudate could act as bioindicators for cultivar-specific sensitivity towards IAA in the rootzone [23]. Dissimilarities in amino acid exudation pattern, in comparison to amino acid composition, were noted and attributed to unknown processes, besides the cell leakage. It was suggested that wheat varieties exposed to similar TRP treatments could possess variations regarding auxin degradation [23], an adaptation strategy to assimilate molecules that counter stress by deploying varied pathways. Accumulation of TRP and histidine (HIS) has been documented during dormancy in red pine needles with falling temperatures [24] and in cold-tolerant *Pinus halepensis* [25]. Increased HIS (ca. 10-fold) in cadmium tolerant species could reduce oxidative stress [26], possibly by regulation of amino acids such as TRP, chelation of Cd, and transport of metal ions, besides plant reproduction and growth [27]. TRP biosynthesis is induced by stresses [28]. Its accumulation competes with Cd availability to plant, decreases Cd transport, and hence imparts tolerance [10].

In controlled conditions (abiotic stress), when the stem cuttings were exposed to treatments (TRP or SER), the trend in titers of TRP continued to be similar to dormant buds when the fate of the buds from the cultivars was vegetative or flowers except in flower samples of natives exposed to controls (water). Here, TRP titers were significantly higher (ca. 2-fold) compared to any sample obtained from native or any treatment, and this could be attributed to the adaptation strategies by natives to counter erratic climatic fluctuations. Treatments (TRP and SER) probably countered a necessary change in TRP titers for flower samples and hence remained on par with the dormant or vegetative samples or metabolized TRP into indoleamines. Locally adapted cultivars did not possess any significant changes in the TRP titers under any given combination. This probably could be due to an adaptation where a different metabolite of TRP, such as nicotinamide in the Kynurenine pathway (not in the scope of this study), could be responsible to counter such abiotic stress conditions or the state of quiescence. Alternatively, it could be a strategical accumulation of downstream indoleamines such as TRM, a molecule known to impart stress tolerance [29].

TRP is known to be involved in protein synthesis, besides being a versatile precursor to indoleamine and kynurenine pathways, where the metabolites assist in stress protection and other vital processes [30]. In animals, this process is tightly regulated by host-microbial interactions where gut commensal bacteria regulate SER synthesis and vice versa. A similar underexplored phenomenon that could probably exist in plants [31], will provide insights into the regulation of indoleamines. For example, sourced cultivars of hazelnuts might harbor a different blend of endophytic microbiome compared to locally adapted ones. Also, foliar application of TRP countered abiotic stress-induced decline of IAA, GA3 and cytokinin titers in wheat shoots, where reduced ABA titers were noted in treated plants [32]. The interplay of IAA, GA3 and cytokinin in shoot tissues of treated plants resulted in an increase in growth rate. The other probable explanation for such differences between locally adapted and sourced cultivars could be the state of quiescence. During dormancy, the locally adapted trees could probably be programmed better than the sourced cultivars that were once conditioned by their then-local climatic conditions and (or) microbiome.

Also, it is known that TRP decarboxylase (TDC) regulates the flux of carbon and nitrogen from the TRP pool into the indolamine pathway, and activation of the enzyme could affect the fate of TRP, depending on the exposure conditions [29]. On the other hand, a two to three-fold increase in the titers of TRP in the flower buds of sourced cultivars exposed to TRP, or control treatments, suggests a regulatory effect exerted by indoleamines such as MEL in the SER treatment, where the concentrations were unaltered [22]. Titers of TRM provided insights into how plants strategize molecule accumulation to adapt quickly and counter the stress successfully. To begin with, the titers of TRM in dormant buds of locally adapted ‘Alex’ and natives, depict an example of how closely related trees find different molecules to counter similar stress. Natives appeared to have chosen TRP accumulation compared to ‘Alex’, where TRM accumulation exemplifies a strategy to counter abiotic stress. Similar examples of TRM accumulation have been reported by our group [33] in a totally unrelated plant species (Hill’s thistle) exposed to different abiotic stress. In several plant species, increased titers TRM accumulation, especially in the reproductive organs, has been noted [29]. It is interesting to note the fold changes in TRM, especially in buds that resulted in flowers, across the cultivars. In ‘Alex’ and natives the fold change appeared to be double and appeared to be unaffected by the treatments (TRP or SER). However, in ‘Delta’ the four-fold change noted in the buds that resulted in flowers when stem cuttings were exposed to controls and TRP but not in SER treatments suggests a regulatory effect of SER on the fate of TRP metabolism, a pattern similar to the titers of TRP. The unavailability of TRM accumulation during dormancy compared to TRP seems to deter ‘Delta’ in allocating resources toward reproduction and utilize indoleamines as a ‘stress buster’ to counter abiotic stress. Preloaded gear of indoleamines in ‘Alex’ that was not affected by treatments could be key in understanding its mitigation strategies to counter abiotic stress. The other possible explanation for improved female flower percentage, besides cold tolerance, could be the fate of TRP other than its contribution to protein synthesis and indoleamine pathway.

Interestingly, the titers of SER analyzed from the treated samples depicted similar trends as that of TRP except that the natives did not accumulate metabolites in the form of SER. Albeit, the stem cuttings from the varieties possessed variations in flowering percentage when exposed to the treatments. Overall, a higher percentage of flowers in stem cuttings exposed to SER followed by TRP and controls suggests the allocation of resources dictated by the environmental conditions. For example, samples from locally adapted ‘Alex’ is probably programmed for such sudden fluctuations and hence analyzed metabolite titers were unaffected but flowering was. This cost appears to be paid off in sourced ‘Delta’ where SER accumulation negatively influenced the flowering percentage by deviating resources towards survival [34]. The pattern of SER titers mirrored TRP and TRM in flower samples of ‘Delta’. Unchanged and unaffected titers of SER in ‘Alex’ demonstrates the importance of tightly regulated TRP metabolism and its fate in unwanted conditions. The titers of NAS in natives, and the strategy to utilize the indoleamine pathway is also noteworthy. The pattern mirroring TRP titers emphasizes varied allocation of resources dictated by the genotype for a similar stress exposure.

Resource allocation was vividly evident when MEL titers were analyzed from the treated flower samples. There is a decline in the accumulation of MEL [17] in flower samples of native hazelnut plants. The decline is seen in the control group and groups treated with TRP and SER. However, there is improved flowering in the reverse order, meaning that the group treated with SER had the best flowering performance, followed by TRP and then the control group. Also, the poor flowering performance of the sourced ’Delta’ hazelnuts across treatments may be due to resource allocation by the plants during abiotic stress. This allocation of resources is reflected in changes in SER and MEL in the control and TRP groups but not in the SER group. Overall, there is a complex relationship between the accumulation of MEL, abiotic stress, and flowering performance in hazelnut plants. More research is needed to fully understand this relationship and its implications for hazelnut production. The hazelnut plants may utilize these titers of MEL to counter stress, suggesting that MEL may play a role in the plant’s response to stress [17]. While the exact mechanisms of the action of indoleamines are yet to be elucidated, the current literature supports the hypothesis of their dual (possibly multiple) modes of action as antioxidants and phytohormones, or even as precursors of metabolites that mimic their action in different physiological conditions.

In conclusion, temperate trees introduced to new locations possess the proper gear to counter abiotic stress and hence pay the cost of compromising flowering percentage. When the stem cuttings of different cultivars were exposed to extreme abiotic stress (−20 °C (winter temperature) to +22 °C (chamber temperature) and 8 h L (winter solstice) to 16 h L (artificial light), with or without ‘stress buster’ support, all buds progressed well but flowering was improved only in treatments, especially for the sourced cultivars. This study exemplifies the importance of TRP and indoleamines in providing short-term and long-term solutions to grow such horticulturally important sourced cultivars which lack TRM during dormancy. A dose-response curve encompassing low doses for TRP and the indoleamines should be constructed for field conditions while considering the importance of a multitude of factors such as microbiome, genetics, and local environmental conditions. Further experiments focusing on the foliar and drenching treatments of these metabolites should be evaluated for resilience in the sourced cultivars in parallel to improved field performances.

## 4. Materials and Methods

### 4.1. Chemicals for UPLC-MS

Analytes chosen for this study TRP, TRM, SER, NAS and MEL belonged to the indoleamine pathway. All standards were analytical grade and purchased from Sigma Aldrich, Canada. All solvents used in the extraction and analytical processes were analytical grade and purchased from Fisher Scientific (Mississauga, ON, Canada).

### 4.2. Plant Material

For the controlled condition experiments, 15-year-old hazelnut (Betulaceae) trees, either native, *Corylus americana* Walter or the selections from locally adapted or sourced *C. avellana* L. were used. Stem cuttings of these hazelnuts experiencing dormancy in field conditions were collected from Simcoe Research Station, University of Guelph at Simcoe, ON (42°51′30″ N, 80°15′56″ W) in February and December of 2018 (I) and December 2019 (II), stored in a freezer held at ca. 2 °C until the experiments commenced.

### 4.3. Bioassays

All experiments were conducted at the Department of Plant Agriculture, University of Guelph, Guelph, ON, in Conviron^®^ growth chambers (Controlled Environments Ltd., Manitoba, MB, Canada) or in the greenhouse. Preliminary experiments mimicking summer-like conditions (abiotic stress) were conducted using 16 L:8 D or 24 L:0 D (photoperiod) and 11, 16, 22 or 30 °C to determine optimum zeitgebers. For a given zeitgeber, stem cuttings from three selections (‘Geneva’, ‘Slate’ and ‘Clark’) were placed in Culture Vessel, PT-12™ (PhytoTechnology Laboratories^®^, Lenexa, KS, Canada) containing ca. 150 mL distilled water that was replenished when needed. Each twig had at least 10 nodes (buds or catkins or combinations). Events occurring within a few hours (ca. 4 h) were regarded as 0.5 d and the data were collected daily until vegetative growth appeared in all the buds. Endpoints of interest included the percent female buds (female ratio), time to full bloom (complete opening of “spiders” stigma), and time to pollen dehiscence. For each selection, there were at least 9 stem cuttings (3 cuttings per vessel) exposed to a given zeitgeber.

**Figure 5:**
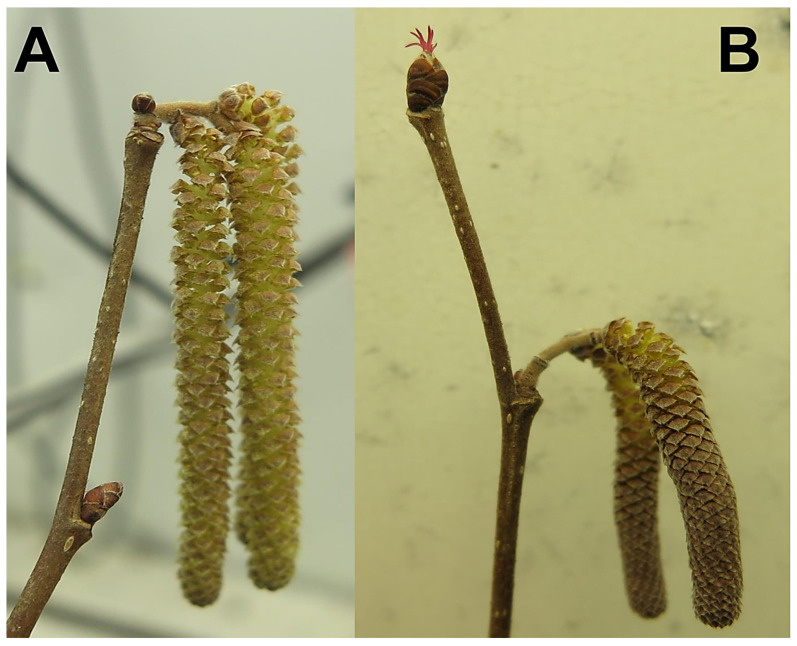
Reproductive organs on dormant stem cutting started dehiscing pollen within 4 h in ‘Geneva’ where the buds remained dormant for a longer period (**A**); Reproductive organs on dormant stem cutting started flowering within 2 days in ‘Slate’ where the catkin remained dormant for a longer period (**B**).

Once a moderate combination of zeitgebers inducing abiotic stress was determined, stem cuttings from either natives or selections of hybrids (locally adapted ‘Alex’ and sourced ‘Delta’) were continuously exposed to either water (control), 100 µM SER or 100 µM TRP dissolved in water. Experiments were set up using the above-described conditions and were replicated within and among chambers and repeated during consecutive years. These experiments were exclusively conducted to obtain tissue for UPLC (Ultra Performance Liquid Chromatography) analyses where analytes such as TRP and its downstream metabolites were assessed. For experiments conducted in 2018–2019, a pooled sample (vegetative and flower bud, Figure 5A,B) was chosen whereas, in 2019–2020, a separate analysis of respective tissue was done. Due to varied temporal responses, catkins (Figure 5A,B) were not chosen for UPLC analyses. Phenological endpoints were recorded daily for the 2019–2020 experiment where percent females were assessed at the end of the experiment.

### 4.4. Sample Preparation

A slightly modified protocol was used to extract phytohormones [35] and is described below. Samples were ground to a fine powder using liquid nitrogen, mortar, and pestle. Later, ca. 100 mg of each tissue was weighed and 0.5 mL of ice-cold (held at −20 °C) extraction buffer composed of 15/4/1 (v/v/v) of methanol/water/formic acid, was added. Samples were briefly vortexed and held at −20 °C for 1 h, centrifuged at 14,000 rpm for 15 min at 4 °C to collect the supernatant, and re-extracted using a similar procedure for 30 min. After pooling, the supernatant from double extractions was then dried under nitrogen gas, reconstituted in 20% methanol and 80% of 0.1% formic acid and filtered through a 0.2 µm centrifuge filter (Millipore Sigma, Oakville, ON, Canada) for 1 min at 14,000 rpm. The flow-through was immediately analyzed on UPLC-MS.

### 4.5. Separation of Metabolites and Metabolite Detection (UPLC-MS)

Phytohormones were separated by reverse phase liquid chromatography (Acquity Classic ultra-performance liquid chromatography system (UPLC); Waters Canada, Mississauga, ON, Canada) by injection of a 5 μL aliquot of sample onto Acquity BEH Column (2.1 × 50 mm, i.d. 2.1 mm, 1.7 μm; Waters). Metabolites were separated with a gradient of solvents A (0.1% formic acid pH 2.7) and B (100% acetonitrile) with initial conditions at 97% A (3% B) increased to 3% A (97% B) over 5 min using an Empower curve of 8. The column temperature was 30 °C and the flow rate was 0.5 mL min^−1^. Metabolite peaks were identified by comparison to standards and quantified by a standard curve generated using a similar separation method and gradient conditions. Phytohormones were detected using a single quadrupole mass spectrometer (Waters, QDa performance model) in single ion recording mode (SIR). TRP, TRM, SER, NAS and MEL were detected in positive mode with a cone voltage of 10, 15, 10, 10 and 15, respectively for mass to charge (m/z) of 205, 144, 177, 220 and 233 respectively. In all cases, the probe temperature was set to 500 °C with a gain of 5; capillary voltage (positive and negative) was set to 0.5 kV. Instrument limits of detection were 6.1 ng/mL, 1.52 ng/mL, 24.4 ng/mL, 6.1 ng/mL and 92.5 pg/mL for TRP, TRM, SER, NAS and MEL, respectively and method detection limits were found to be 0.407 μg/g, 0.407 μg/g, 1.62 μg/g, 0.407 μg/g and 6.17 ng/g, respectively. The linear range for TRP, TRM, SER, NAS and MEL was 24.4 ng/mL–25 μg/mL, 6.1 ng/mL–6.25 μg/mL, 97.7 ng/mL–25 μg/mL, 24.4 ng/mL–25 μg/mL and 38.1 pg/mL–6.25 μg/mL respectively [36].

### 4.6. Statistical Analyses

Preliminary bioassay experiments were a completely randomized design, with zeitgebers being the main factors of interest whereas, for the 2019–2020 experiments, treatments (SER or TRP) and selections (‘Alex’, ‘Delta’ or native hazelnuts) and kind of bud (vegetative or flower) were the main factors. Data were analyzed using Proc GLIMMIX in SAS^®^ Studio (SAS Institute, Cary, NC, USA). Residuals were used to verify the assumptions of normal error distribution and constant variance. For main factors or interactions, if the means were significantly different, they were separated using Tukey’s test (α = 0.05). In the phytohormone analyses, there were 3 replicates per bud type per selection or natives (flowering and vegetative). Source of hazelnuts, kind of bud and treatment (SER or TRP) for hazelnuts, whereas concentrations of SER or TRP were considered as fixed factors and the metabolite responses (µg/g FW) were subjected to ANOVA using PROC GLIMMIX in SAS^®^ Studio (SAS Institute Inc., Cary, NC, USA). For all responses, the normal distribution and constant variance assumptions on the error terms were verified by examining the residuals. When the effects were significant, least square (LS) means were separated at α = 0.05 level. Mean ± SEM (µg/g FW) responses per each metabolite are presented in a graphical format.

## Data Availability

Not applicable.
